# The role of aging in cancer

**DOI:** 10.1002/1878-0261.13302

**Published:** 2022-09-20

**Authors:** Aaron Havas, Shanshan Yin, P. D. Adams

**Affiliations:** ^1^ Sanford Burnham Prebys Medical Discovery Institute La Jolla CA USA

## Abstract

Many cancers show an increase in incidence with age, and age is the biggest single risk factor for many cancers. However, the molecular basis of this relationship is poorly understood. Through a collection of review articles, our thematic issue discusses the link between aging and cancer in aspects including somatic mutations, proteostasis, mitochondria, metabolism, senescence, epigenetic regulation, immune regulation, DNA damage, and telomere function.

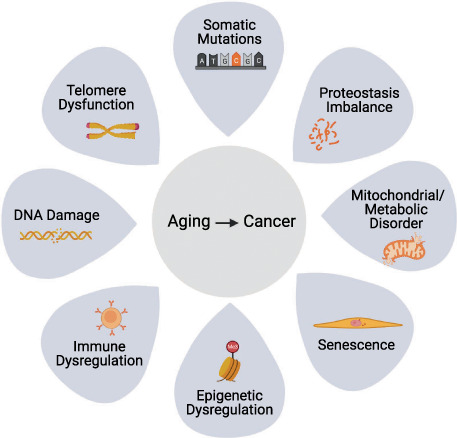

**Abbreviations**
CRC: colorectal cancer
NAFLD: non‐alcoholic fatty liver disease
SASP: senescence‐associated secretory phenotype

Many cancers show a striking increase in incidence with age, and age is the biggest single risk factor for many cancers. Moreover, pro‐longevity interventions that extend lifespan also tend to suppress the incidence of cancer, underscoring the tight relationship between aging and cancer [1]. However, the molecular basis of this relationship, and why the incidence of cancer increases with age, is poorly understood.

## 1 Cancer in the elderly is not simply a reflection of the time taken to accumulate oncogenic mutations

Genetic mutations are critical drivers of most cancers [2]. Oncogenic mutations accumulate with age in many tissues, including blood [3, 4, 5, 6], brain [7], skin [8], esophagus [9, 10], colon [11], and liver [12, 13]. Presaging these recent discoveries, Doll and Armitage [14], as well as Nordling [15], originally proposed that the age dependence of cancer can be explained by the time taken to accumulate multiple discrete events required for onset of disease. Specifically, in their seminal analysis of the age dependence of cancer, Doll and Armitage stated that “The data can be said to accord with the theory that 6 or 7 successive changes in the cell are necessary before cancer appears as a clinical entity”. Subsequently, there has been a tendency to assume that the age dependence of cancer results from the time taken to accumulate the requisite genetic alterations, in the appropriate cells. However, significantly, Doll and Armitage did not assume that the “successive changes” are necessarily genetic alterations [14]. Indeed, several lines of evidence argue against the idea that time taken to accumulate genetic mutations is the sole reason for age dependence of cancer.

At first glance, the so‐called “Vogelgram” proposed by Vogelstein and coworkers [16] supports the notion that the time taken to accumulate oncogenic mutations underpins the age dependence of cancer. This paradigmatic model schematizes the initiation and progression of colorectal cancer (CRC) through an idealized histological sequence linked to acquisition of recurrent genetic alterations. Although this model was transformative for conceptualizing the origins and progression of CRC and then other cancers, the Vogelgram is primarily a model of histological progression, not time‐dependent progression. Indeed, it has been estimated by Vogelstein and coworkers that the entire progression from normal intestinal epithelium to CRC takes approximately 28 years [17]. CRC has an average age of diagnosis at 68 or 72 years (men and women, respectively; www.cancer.org), so the Vogelgram does not adequately explain the age dependence of CRC. More recent models have proposed a punctuated model of cancer evolution, whereby one or a few genetic catastrophes, encompassing many simultaneous events, are key in genetic evolution of cancer [18, 19, 20]. These models also do not explain why genetic catastrophe should be a relatively late‐life event.

Furthermore, contrary to the seeming inevitability of genetic and histological progression conveyed by the Vogelgram, more recent sequencing studies, for example, of benign human nevi, normal human skin, and endometrium, have revealed that oncogenic alterations are surprisingly well tolerated by normal adult human tissues [8, 21, 22]; why these events are seemingly less well tolerated in aged tissues, leading to cancer, is not known.

Contrary to the view of stochastic clonal expansion of tumor cells in a sea of otherwise normal cells, some studies in mice suggest that old tissues are inherently at risk of cancer, compared to young tissues. Non‐alcoholic fatty liver disease (NAFLD) is a chronic liver disease that encompasses a progressive range of disorders of increasing severity and risk of hepatocellular carcinoma, from benign fatty liver (steatosis) to inflammatory non‐alcoholic steatohepatitis, fibrosis, and cirrhosis [23, 24, 25]. Old mice are more prone to high‐fat diet‐induced steatohepatitis than young mice [26, 27, 28, 29]. Old mice also show more fibrosis after chemical injury by CCl_4_ [30] and are more susceptible to alcohol‐induced liver injury, inflammation, oxidative stress, and fibrosis than young mice [31]. The aged liver promotes the clonogenic growth of transplanted normal or pre‐neoplastic hepatocytes [32, 33]. Together these results suggest that aged hepatocytes and/or the aged microenvironment are inherently pre‐disposed to tumorigenesis.

Finally, many lifestyle and environmental risk factors for cancer, such as obesity and lack of exercise, are currently not readily explained by genetic progression models [34]. Conversely, whether pro‐longevity interventions that characteristically also suppress cancer, for example, calorie restriction, intermittent fasting, drugs such as metformin and rapamycin, along with genetic deficiency of the growth hormone and insulin‐like growth factor pathways, act primarily by reinforcing genetic integrity is not known [35].

## 2 Other potential causes of age‐associated cancer

Tomasetti and Vogelstein recently proposed that mutations acquired through random errors during normal DNA replication play a major role in origin of cancer, together with other events triggered by environmental and lifestyle factors and heredity [36, 37]. They also proposed that replication errors can explain the extreme variation in cancer incidence across different tissues, because the lifetime number of stem cell divisions, that is, rounds of DNA replication, correlates with the lifetime risk of cancer in that tissue. However, these studies did not themselves address whether the oncogenic consequence of stem cell divisions is genetic (i.e., DNA mutations) or non‐genetic (e.g., epigenetic, inflammation, metabolic changes or alterations to the stem cell niche). Interestingly, most other common diseases of aging, Alzheimer's, cardiovascular disease, type II diabetes, etc., are not documented to be caused by somatically acquired genetic alterations [38]; instead, protein misfolding, metabolic changes, and other non‐genetic defects tend to take center stage in these diseases. And, while cancer differs from these other diseases in important respects (i.e., clonal expansion), it is reasonable to propose that some shared molecular processes underlie cancer and other diseases of aging [1].

**Fig. 1:**
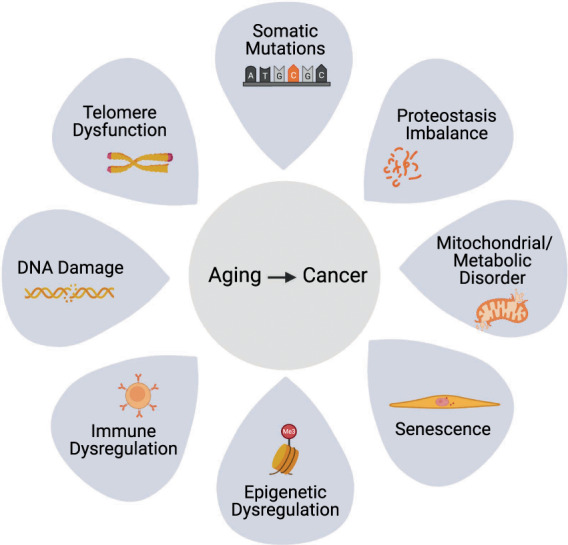
The link between aging and cancer. The potential causes of age‐associated cancer include accumulated somatic mutations, imbalanced proteostasis, mitochondrial or metabolic disorders, cellular senescence, epigenetic dysregulation, immune dysregulation, DNA damage, and telomere dysfunction.

Candidate drivers of cancer linked to aging include the “hallmarks of aging” [35, 38], known to be dysregulated with age in diverse tissues and organisms. As well as genetic mutations, these hallmarks include changes to mitochondria, the epigenome, metabolome, accumulation of senescent cells, inflammation, and immune changes. In some respects, these hallmarks of aging parallel the hallmarks of cancer, in that both reflect accumulation of molecular, cellular, and tissue damage [39, 40], further supporting the idea that multiple age‐associated changes within normal tissue can be drivers of cancer (Fig. 1).

Even a consideration of genetic mutations alone raises important questions regarding the age dependence of cancer. As noted above, genetic mutations are critical drivers of most cancers [2], and genetic mutations accumulate with age in many tissues, including blood [3, 4, 5, 6], brain [7], skin [8], esophagus [9, 10], colon [11], and liver [12, 13]. However, as reviewed by James DeGregori and colleagues, the potential response of cells and tissues to accumulated mutations in tissues is complex, ranging from inflammation, suppression of growth or elimination of mutated cells, competitive interactions with neighboring normal cells, tissue functional decline and, in some cases, cancer [41].

Protein homeostasis—or proteostasis—is the process by which the cell's complement of proteins is maintained in working order, by balancing and controlling protein synthesis and degradation, protein folding, and protein complex assembly and disassembly. The integrity of this complex process declines with age and is thought to contribute to aging in many ways [42]. For example, age‐dependent accumulation of misfolded proteins promotes inflammation, a contributor to numerous age‐related diseases including cancer [43]. Autophagy is a central mechanism for degradation and recycling of organelles and proteins [44]. Dysregulated autophagy is implicated in both aging and cancer, as reviewed by Masashi Narita and colleagues [45].

Mitochondria play key roles in oxidative phosphorylation, redox balance, apoptosis, and numerous biosynthetic pathways. Declining mitochondrial function is a hallmark of aging and has also been proposed as a driver of aging phenotypes and disease, including cancer [46, 47]. Age‐associated mitochondria dysfunction is, in part, due to accumulation of mutations within the mitochondrial genome. The contribution of such mutations to aging and cancer is reviewed by Laura Greaves and colleagues [48].

Emerging evidence links the major metabolic diseases of aging, for example, metabolic syndrome, type 2 diabetes, and obesity, to increased risk of cancer. This is most obvious in the case of liver cancer, where NAFLD is both the liver manifestation of metabolic syndrome, linked to diabetes and obesity, and also a risk factor for liver cancer [23, 24, 25]. More specifically, age‐dependent metabolic changes are well documented and some, for example, nicotinamide adenine dinucleotide depletion, induce a pseudohypoxic state and Warburg reprogramming similar to cancer [49]. At least some of these changes are likely causative of cancer. Ana Gomes and colleagues review how age‐induced metabolic programming alters tissues to promote an environment that is conducive to transformation, and also to suppress immune surveillance and anti‐tumor host defenses [50].

Cellular senescence is caused by a range of cellular stresses and characterized by an irreversible proliferation arrest and a potent pro‐inflammatory phenotype, the senescence‐associated secretory phenotype (SASP) [51]. Senescence‐associated proliferation arrest and SASP cooperate in tumor suppression, by arresting proliferation of damaged pre‐malignant cells and promoting immune clearance. Although senescence is acutely tumor suppressive, over the longer term, as a source of chronic inflammation, SASP also promotes tissue aging and disease, as reviewed by Rugang Zhang and colleagues [52] and Naoko Ohtani and colleagues [53].

Cancer initiation and progression depends on diverse modes of innate and adaptive immune dysregulation, including chronic inflammation, immune tolerance, and recruitment of immunosuppressive immune cells [54]. For example, expression of immune checkpoint inhibitors, for example, PDL1 and PDL2, on tumor cells confers resistance to cytotoxic T cells [55]. Cell surface‐expressed PDL1 and PDL2 interact with PD1 on T cells, thereby downregulating T cell activity. Similarly, CD80 and CD86 interact with T cell CTLA4 and also downregulate T cell activity. Expression of these immune checkpoint inhibitors on tumor cells confers resistance to cytotoxic T cells [55]. Aging is generally associated with altered immune function and chronic inflammation and this is thought to promote diseases of aging, for example, through so‐called “inflamm‐aging” [56].

DNA repair is essential for maintenance of DNA integrity and function. Deficiencies in DNA repair have been suggested to contribute to aging and cancer in two ways: First, through accumulation of mutations that affect genome function, and second, through unrepaired DNA lesions that activate diverse cell stress responses, from inflammation to senescence to apoptosis [57]. Important recent studies showed that rates of mutation accumulation track inversely with lifespan, suggesting that DNA repair mechanisms have evolved in line with lifespan and that genome mutational load limits lifespan [58]. As reviewed by Raul Mostoslavsky and coworkers, targeting DNA repair pathways is a candidate intervention for both healthy and cancer therapy [59].

Dysfunctional telomeres can be pro‐aging and tumor suppressive by limiting cell proliferative capacity. On the other hand, dysfunctional telomeres, reminiscent of DNA double‐strand breaks, can also promote genome DNA recombination events, genome instability, and cancer [60]. Lea Harrington and colleagues discuss this dual role of telomeres in aging and cancer, including as targets for therapeutic interventions and potential cross‐over of healthy aging interventions and cancer prevention [61].

## 3 Reducing the burden of cancer

On December 23, 1971, President Nixon signed into law the United States' National Cancer Act, the so‐called “War on Cancer”. Between 1971 and 2019, a period of nearly 50 years, death rates from all cancers declined by 27% [62]. Moreover, progress has been uneven demographically and across cancer types. Although a huge amount has been learned and there have been profound therapeutic advances, as illustrated by much improved treatments for chronic myeloid leukemia, melanoma and advances in immunotherapy, the overall impact on death rates cannot be seen as an unqualified success story.

For virtually all cancers, the chances of survival increase with earlier detection. For some cancers, such as pancreatic cancer, the low survival rates can be largely attributed to challenges of early detection. Since most adult human cancers are diseases of aging, we can reason that understanding the role of aging in initiation and development of cancer is a key to risk assessment and early detection. Unfortunately, the role of aging in cancer has not been a central pillar of research in the War on Cancer over the last 50 years. The articles in this volume illustrate the diversity of mechanisms by which aging contributes to cancer, and provide glimpses of how an understanding of aging and cancer can potentially transform risk assessment, early detection, and even prevention.

## 4 Conclusions

In a small collection of this size, it is not possible to cover all topics, and there are some notable omissions. Stem cells occupy a special place in thinking about aging as cancer, as relevant targets for both tissue exhaustion in aging and dysregulated cell renewal in cancer [63]. Age‐associated epigenetic changes are well characterized in many cells and tissues and are also thought to promote cancer [64, 65, 66, 67]. Even with these gaps in the story, this series of articles hopefully conveys the complexity of mechanisms underlying aging and cancer and helps to further invigorate research in this fascinating and important topic.

## Conflict of interest

The authors declare no conflict of interest.

## Author contributions

All authors contributed equally to this article.
